# Methodological issues when using face prototypes: A case study on the Faceaurus dataset

**DOI:** 10.1017/ehs.2022.25

**Published:** 2022-10-28

**Authors:** Jeanne Bovet, Arnaud Tognetti, Thomas V. Pollet

**Affiliations:** 1 Department of Psychology, Faculty of Health and Life Sciences, Northumbria University, Newcastle upon Tyne, UK; 2 Department of Clinical Neuroscience, Karolinska Institutet, Stockholm, Sweden

**Keywords:** face perception, facial stimuli, external validity, pseudoreplication, personality

## Abstract

Prototype faces, created by averaging faces from several individuals sharing a common characteristic (for example a certain personality trait), can be used for highly informative experimental designs in face research. Although the facial prototype method is both ingenious and useful, we argue that its implementation is associated with three major issues: lack of external validity and non-independence of the units of information, both aggravated by a lack of transparency regarding the methods used and their limitations. Here, we describe these limitations and illustrate our claims with a systematic review of studies creating facial stimuli using the prototypes dataset ‘Faceaurus’. We then propose some solutions that can eliminate or reduce these problems. We provide recommendations for future research employing this method on how to produce more generalisable and replicable results.

## Graphical abstract

**Figure:**
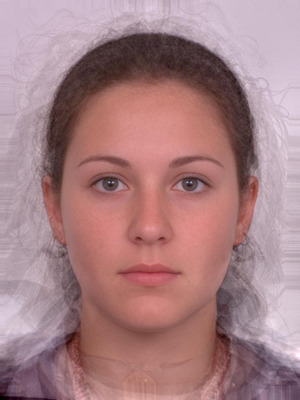

**Social media summary:** Are personality traits visible in faces? Methodological issues when using face prototypes in face research.

## Introduction

Face perception plays a critical role in social interactions and has a considerable impact on human behaviour. It is thus unsurprising that a great deal of scientific research is dedicated to discovering the variety of effects that facial appearance has upon social functioning (Re & Rule, 2016). A subfield of this research focuses on the relationships between faces and personality, including evolutionary behavioural sciences (Brown & Sacco, 2016; Marcinkowska et al., 2016). A major and robust finding of this literature is that there is consensus on inferring personality characteristics from facial appearance: independent observers agree about a person's personality trait based solely on images of their face (Cogsdill et al., 2014; Todorov et al., 2015; Walker & Vetter, 2016), although observer characteristics influence person perception as well (Hehman et al., 2017). One interesting question naturally arising from this observation concerns the *accuracy* of these concordant judgements: can we trust such perceptions, or at least, is there a ‘kernel of truth’ to judgements of personality made from faces? Although a lot of research has been conducted to address this question (see Bond et al., 1994; Zebrowitz & Collins, 1997 for some of the first examples of this line of research), there still is no definitive answer (Walker & Vetter, 2016). This subfield of face research investigating how faces reflect personality and social characteristics (including the Big Five, the Dark Triad, Sociosexuality, Dominance, Trustworthiness, Cooperativeness, Intelligence, etc.) uses a variety of methods (e.g. Bonnefon et al., 2017; Oosterhof & Todorov, 2008; Todorov et al., 2015). One key method relies on the creation of ‘prototype’ faces (sometimes called ‘average’ or ‘composite’ faces). Prototype faces are used to extract the defining characteristics of a group while losing the characteristics that make each face look individual (Penton-Voak et al., 2006). Francis Galton first developed facial prototyping more than 140 years ago, by making multiple exposure photographic images of several faces after aligning the eye positions (Galton, 1878). More recently, computer graphic methods were developed to create realistic prototype faces (Tiddeman et al., 2001). A typical procedure is as follows. The first step to this method is to measure the trait of interest (e.g. extraversion) in a sample of participants. Next, the sample is separated into two groups: one group with participants scoring low on the trait of interest (e.g. low extraversion scores) and one group with the participants scoring high on the same scale (e.g. high extraversion scores). Two prototype faces are then created by averaging all of the faces in each group (Tiddeman et al., 2001). The resulting prototypes are either directly used as stimuli (see Alper et al., 2021a or Moore et al., 2013 for some examples) or their facial information is used to transform another set of faces (for example see Brown et al., 2019). Finally, the facial stimuli (prototypes or transformed faces) are evaluated by a new sample of participants (the ‘observers’), typically in a rating or forced-choice task. The procedural details of the observation task vary between studies, but generally, the observers are asked to make a judgement on the same trait used to create the prototypes (extraversion for our example) to measure the accuracy of their judgement, or another trait (for example, attractiveness) to investigate if people use facial information to adjust their preferences or behaviour in a way that is predicted by the trait captured in the facial stimulus. For example, people are expected to rate a facial stimulus reflecting ‘positive’ characteristics, such as high agreeableness, as attractive as opposed to one with ‘negative’ characteristics.

Although the prototype method is quite ingenious and promising, the way it is commonly used is problematic for several reasons (notably, a study using this method was recently criticised; see DeBruine, 2020). Here, we argue that the current implementation of the prototype method gives rise to the issues of lack of external validity and non-independence of units of information. External validity is widely acknowledged to be an important issue (Findley et al., 2021; Lesko et al., 2017; Steckler & McLeroy, 2008): can effects observed in one study be generalised to different measures, people, settings and times? Non-independence of units of information is a well-known problem as well, although seemingly not always well understood or detected. Here a ‘unit of information’ can refer to an observation, some facial information or the result of a study. Most statistical tests assume that observations are independent (Quinn & Keough, 2002: 2), and if we do not account for dependence in the data, we might draw erroneous conclusions (Kruskal, 1988). Similarly, in incremental research, each new study needs to introduce new data independent from previous data points, and hidden dependence across studies might result in an overestimation of our confidence in the replicability of the results.

The two issues of external validity and non-independence are sometimes grouped under the name of ‘pseudoreplication’ (McGregor, 2000). While pseudoreplication is well understood in animal behaviour research (Freeberg & Lucas, 2009; Hurlbert, 1984; Kroodsma et al., 2001; McGregor, 2000; Waller et al., 2013), there appears to be a less widespread awareness of this issue in other fields of the behavioural sciences, including evolutionary psychology (but see, Lazic, 2010; Ramírez et al., 2000; Winter, 2011). We argue that this lack of awareness of the methodological limitations is reflected in the publications using the ‘prototype method’ and is worsened by a lack of transparency regarding the details of the methods used. The purpose of our paper is thus to review the three issues of lack of external validity, non-independence of units of information and lack of transparency in research relying on the aforementioned ‘prototype method’.

### This study

We evaluate the issues of external validity and non-independence of units of information in studies using the facial prototype method to explore personality judgement made from faces. Concomitantly, we evaluate a third aspect of this literature, namely the transparency regarding the stimuli methods used and its limitations.

While reviewing the literature using the prototype method, we noticed a series of papers using the same dataset of prototypes, namely the Faceaurus dataset (Holtzman, 2011), including recent publications (e.g. Alper et al., 2021b), and this dataset is also covered in a textbook (Bereczkei, 2017). Holtzman (2011) was the first to use the prototype method to see if the ‘Dark Triad’ of personality, i.e. Narcissism, Machiavellianism and Psychopathy, could be detected in neutral faces. While the focus of the research paper was on the Dark Triad, Holtzman also made a dataset publicly available (Holtzman, 2018), which next to the Dark triad personality traits included prototypes scoring high and low on Big Five factors, on each of the 30 facets of the Big Five, as well as prototypes based on measures of arrogance, ingenuity, intrasexual competition, long- and short-term mating orientation and attractiveness, and on schizoid, antisocial and obsessive–compulsive personality disorders. This dataset includes a total of 94 male prototypes (two prototypes for each of the 47 traits) derived from 33 male participants and 94 female prototypes derived from 48 female participants. As such, the number of prototypes (*N* = 188) is considerably higher than the number of individuals in the original sample used to create these prototypes (*N* = 81).

Given that we found that this dataset was widely used, we decided to systematically review all the documents using the Faceaurus, and to use this corpus as an example of the issues in this field. Importantly, the methodological issues revealed in our study are not limited to the papers using the Faceaurus, and some of these issues (when not all of them), can be found in studies using other datasets. However, we believe that focussing on one particular dataset makes for a good case study.

## Methods

We retrieved all documents citing Holtzman (2011) in Google scholar, which returns lists of citing references more complete than those of Web of Science and Scopus (Lasda Bergman, 2012). This dataset was created on 16 August 2021. On this date, Google Scholar gave 95 results for citations of Holtzman (2011). Each of the three authors read and coded a third of these 95 references. Any ambiguous cases were flagged and discussed with the other authors until an agreement was reached. The list of the variables used to code these outputs is presented in Table 1 and the data is available at https://osf.io/t2kz3. The two necessary criteria to include a publication in the final dataset were: (1) it was published in a peer-reviewed journal; and (2) it used prototype faces from the Faceaurus dataset.

**Table 1.** List of the variables used to code the publications in our dataset

Variable name	Variable description	Variable type
Prototype faces	Are prototype faces used as stimuli?	Binary
Faceaurus	Do (some of) the prototype faces used come from the Faceaurus dataset?	Binary
Trait used for the prototype	Which personality trait(s) was(were) used to create the prototypes (for example ‘Extraversion’)	One binary variable per trait
Mention *N* sample	Was the original sample size (i.e. the full sample from which the faces used to create the prototypes were drawn) specified in the paper?	Binary
Mention *N* prototype	Was the number of faces used to create each prototype specified in the paper?	Binary
*N* male sample	Number of men in the original full sample used to create the prototype faces (33 if the dataset used was Faceaurus)	Continuous
*N* female sample	Number of women in the original full sample used to create the prototype faces (48 if the dataset used was Faceaurus)	Continuous
*N* per male prototype	Number of male faces used for each male prototype face (10 if the dataset used was Faceaurus)	Continuous
*N* per female prototype	Number of female faces used for each female prototype face (10 if the dataset used was Faceaurus)	Continuous
Individual faces	Are individual faces used in addition to the prototype faces?	Binary
Stimuli type	What kind of stimuli is shown to observers (e.g. prototypes or other faces transformed using prototypes)	Categorical
Evaluation method	The method used by the observers to evaluate the facial stimuli (e.g. individual ratings; forced choice; extended forced choice)	Categorical
Evaluated trait	Trait(s) that observers had to evaluate the stimuli on (e.g. Extraversion or attractiveness)	Categorical
*N* observers	Number of observers evaluating the facial stimuli	Continuous
Acknowledgement of limitations	Did the authors acknowledge any limitations linked to the stimuli set (e.g. small original sample size or limited external validity)	Binary
Replication	Did the authors use another stimuli dataset in addition to the Faceaurus?	Binary

First, we examine the issue of external validity of the Faceaurus dataset and its consequences for the studies in our systematic review. Second, we discuss the issue of non-independence of units of information at different levels, including at the stimuli production, study design and research field levels, still using the final dataset of our systematic review. Third, while reporting our findings on these two issues of external validity and non-independence, we describe the level of transparency regarding the stimuli method used and its limitations.

## Results

### Description of our dataset

Including Holtzman's (2011) paper itself, we found 25 outputs using the Faceaurus dataset, including two theses and two preprints, which left us with 21 papers published in peer-reviewed journals as our final dataset.

### External validity

The goal of the majority of behavioural research is to draw conclusions about a specific population of individuals by examining a sample of individuals from this population (Simons et al., 2017). Scientists should thus seek the largest and most representative samples they can achieve in order to increase the confidence of extrapolating findings from their sample to the population. Although most face research scientists seem to be aware of the importance of external validity in the samples of ‘observers’ they use (as seen in the use of relatively large sample sizes of participants evaluating the facial stimuli), the external validity of the facial prototypes used as stimuli is largely disregarded. The external validity of prototypes is entirely conditional on the external validity of the original sample used to create them. Here, we argue that these original samples, and thus the resulting prototypes, are rarely representative of the groups they are meant to represent.

#### Size, range and representativity of the full sample of faces

Although the studies in our review relied on relatively large samples of observers (*M* = 602; SD = 686), the size of the original sample to create the stimuli was much smaller. Indeed, all the papers included in our final dataset (21^1^ out of 21) exclusively used the prototypes from the Faceaurus dataset, which were created from a small original sample: just 33 men and 48 women.^2^

The main issue with such a limited sample size is range restriction. Simply put, it is unlikely that with a small number of men (*N* = 33), we will have respondents with very high scores of psychopathy, for example. This is problematic, as studies using the Faceaurus typically aim to make inferences about ‘psychopathic’ behaviour. For example, Lyons et al. (2015: 157) write: ‘Thus, if information about these types of behaviours can be gleaned from the facial structure, it would be adaptive to avoid these men due to risk of violent injury, or in extreme cases, death’. However, these violent men may be nowhere to be found in the stimuli set. A study with 1,510 Belgian participants (48% men) argued that 43 respondents (2.9%; 2 SD from the mean) scored extremely high on psychopathy (Gordts et al., 2017). In a sample of 33 men, we can thus expect around one or two male participants to show such an extreme score. We can generate high and low prototypes on a trait which significantly differ in appearance, but it is unlikely that they will represent the *extreme* of a trait. Thus, while such prototypes are argued to represent, for example, psychopathy, there were (probably) no psychopaths in the stimulus sample to begin with. The same logic applies to other traits collected in the Faceaurus: with small samples the extremes of any given trait of the population at large are probably not represented. If we take as an arbitrary heuristic that 2.5% of the sample score extremely, then around one male and female participant per trait is expected to score extremely high or low on any given trait in the Faceaurus. For some traits, this will imply that there are no extremes included in the generation of any given prototype. Because a sample of population extremes might look very different than one using the top or bottom 10 from a sample of 33 men and 48 women, there is also no guarantee that the features will correspond between a prototype generated from ‘true’ psychopaths drawn from a representative male population vs. a prototype derived from 33 men. This issue is accentuated by the fact that the original sample for the Faceaurus consisted of students, as the range of personality traits in students is expected to be more restricted than a sample of the same size taken from the general population. In sum, for studies using the Faceaurus, it is important to acknowledge its limited range – the true, extreme, trait of interest (e.g. psychopathy) might not be captured at all. Only one publication (one out of 21) discussed the restricted range of personality scores in the original sample (Lyons & Blanchard, 2016). Surprisingly (and worryingly), none of the publications in our dataset (zero out of 21) mentioned the small size of the full original sample as a limitation. Moreover, only one of the publications in our literature review (one out of 21, namely Holtzman 2011) reported the size of the full original sample from which the faces used for the prototypes were drawn.

#### Size of the subsamples of faces

Independently of the size, range or representativity of the full original sample, the external validity of the prototypes would be limited by the even smaller subsamples of faces selected to create the prototypes. All the studies in our review used prototypes from the Faceaurus dataset, which are made by averaging 10 individual faces (e.g. the faces of the 10 individuals with the higher scores on extraversion in the full original sample). A sample size of 10 would be considered insufficient for the vast majority of behavioural studies, and we argue that there is no valid reason why a sample used to create facial prototypes should be an exception. Importantly, this issue would remain even if the full original sample size was larger, or if the sampling method was intentionally targeting individuals scoring extremely high or low on a trait (by recruiting clinical populations for example), as a sample of 10 individuals to generate prototypes remains too small to be representative of any group. None of the publications in our dataset (zero out of 21) mentioned the small size of the subsamples as a limitation, and only two-thirds of them (14 out of 21) reported the number of faces used per prototype (i.e. per group). The remaining seven publications did refer to at least one previous publication mentioning this information (for example, Holtzman 2011 or one of their own previous publications), which makes this critical information technically available but more difficult to retrieve or evaluate.

To conclude, although the lack of external validity of the prototypes from the Faceaurus is both manifest and problematic, this was never mentioned as a limitation in the publications in our dataset. This is intriguing, as external validity was something acknowledged in several of these publications in reference to the sample of observers – which can be seen through the use of large and diverse samples of observers and direct mentions in the text (see Lyons & Blanchard, 2016: 43, for example) – but never regarding the stimuli set.

### Non-independence of units of information

In the above, we focused on the lack of external validity of the prototypes in the Faceaurus dataset, resulting from the limitations of the original sample which was used to generate them. Here, we argue that the way these non-representative stimuli are created and used generates dependence of units of information at different levels. If we do not account for dependence in the units of information, then we will overestimate the degrees of freedom which could lead to erroneous conclusions (Kruskal, 1988). The degrees of freedom in studies using prototypes are constrained by both the stimuli and the observers, i.e. we can have many observers, but they cannot be treated as independent units of information, as the same stimuli (or different but non-independent stimuli) are used. At a higher level, in incremental research, each new study needs to contribute new data, independent from previous studies, in order to increase our confidence in the robustness of a scientific result. Here, we argue that the sources of dependence in studies using the prototype method are the following: (1) the studies use a single prototype per group; (2) the same individual faces make up different prototypes; (3) the same prototype is used to produce different stimuli; and (4) the same stimuli are used across different studies.

#### One prototype per group

In all of the 21 publications included in our final dataset, only one prototype per group (e.g. high extraversion group) was used (the one available in the Faceaurus dataset) to test hypotheses about the whole population itself (e.g. extravert individuals). This creates dependence in the observations and constitutes a case of ‘simple pseudoreplication’ (McGregor, 2000). To illustrate the concept of pseudoreplication with an obvious example (borrowed from McGregor and expanded), imagine you wanted to test whether the height of men differed from that of women. You measure the height of one man six times and of one woman six times. You then perform a *t*-test using the number of measurements made, *N* = 12. This is incorrect, as the true number of statistically independent replicates for the test is two and not 12 since there was only one man and one woman. This is a case of pseudoreplication. Now, instead of measuring the height of your subjects six times yourself, let us assume that you ask a sample of 200 participants to come into the lab and measure the height of the two same subjects (each participant measures the height of the woman once and the height of the man once). Now that you have 200 different participants measuring height, your height measurement might be more precise, but the number of statistically independent replicates is still two (one man and one woman). This is because the question you wished to answer was not whether your two subjects differed in height, but the more general question of whether men (in the population) were taller than women (in the population). Assuming that the sample size is 200 per measurement would be committing pseudoreplication. Studies using the ‘prototype method’ do not use one single individual as stimulus, but they do use one single prototype. Although a composite stimulus (e.g. a prototype made of several faces scoring high on extraversion) is better than a single individual face used as a stimulus (e.g. the face of one single individual with a high extraversion score), this approach still constitutes simple pseudoreplication, because only one stimulus is used to make inferences to a whole population (e.g. all extraverts). In our height example, it would correspond to creating a ‘prototype body’ by averaging the body shapes from 15 men and another prototype body averaged from 15 women, then having each prototype body measured once by 200 participants. The height difference will be more accurate than when using only one individual man and one woman, but the sample size for each measurement still is not *N* = 200. Note that this is an issue only because the research question is not about the perception of these two specific composite stimuli, but about the broader population they represent (e.g. all extraverts). As such, in this case, the non-independence of observations is intrinsically linked to the issue of external validity discussed above. None of the publications in our dataset (zero out of 21) discussed the risk of pseudoreplication or non-independence of observations when using a single prototype per group.

#### The same prototypes feature in different stimuli

In some publications, the prototypes were directly used as the stimuli presented to the participants (see Alper et al., 2021a for an example). In other publications, more than one facial stimulus was created for each group by using the low and high prototypes to transform a new set of faces (see Brown et al., 2019 for an example). In order to achieve this a new set of individual faces (sometimes called the ‘bases’) was transformed (or ‘morphed’) with one single pair of prototypes from the Faceaurus. As a result, there was a new pair of faces for each base, comprising one ‘high’ and one ‘low’ version, which were used as stimuli. However, these different facial stimuli were always created using the *same* single prototype per group, meaning that the facial stimuli were not independent of one another. Indeed, this technique would be comparable with presenting the same prototype several times to the participants, but each time with a different background colour: although the overall facial information differed from one pair to another, the variable of interest (in this case the facial information linked to the personality trait of interest) was exactly the same for all the pairs of stimuli. As a result, the same difference in facial information was evaluated several times by each observer (i.e. repeated measures design). The base faces (the individual faces which were transformed to create the new set of stimuli) might of course have impacted the observers’ perception, but this should only shift the baseline, and the difference between the two faces of each pair should remain unchanged. To be fair, in the studies in our dataset, the observers’ responses were aggregated at the prototype level (e.g. averaging the ratings for all the stimuli transformed with the same prototype), removing the statistical issue of non-independence of the observations. Nonetheless, we believe that the used methodology remains a concern, as it gives the false reassuring impression that several independent stimuli were used, thereby inflating the confidence that one can have in the results. This is particularly the case when the description of the method used to create the stimuli is either incomplete or unclear, which was often the case in our dataset.

#### The same individual faces make up different prototypes

**Figure 1.**
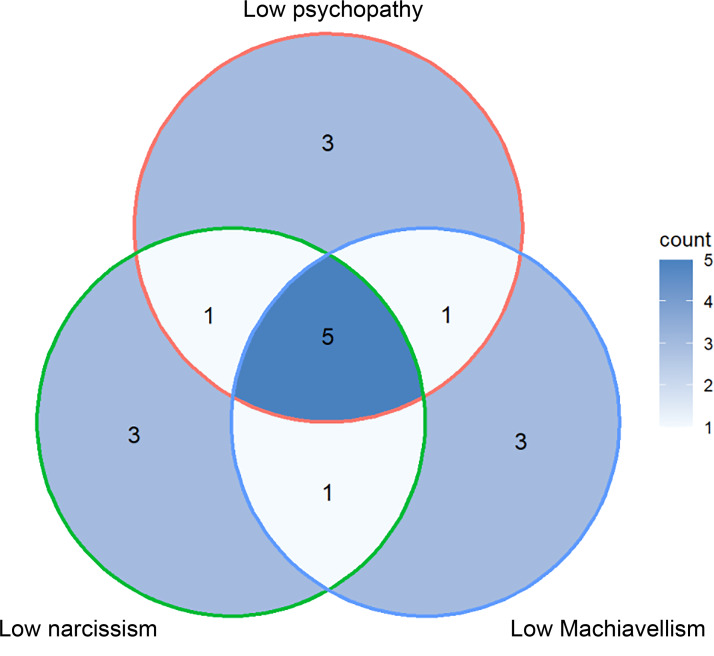
Venn diagram for the individual faces used to create the Low Dark Triad male prototypes in Faceaurus (*N* = 33 men). Prototypes consisted of 10 faces each. The three prototypes ‘low psychopathy’, ‘low narcissism’ and ‘low Machiavellism’ have five faces in common. The ‘low psychopathy’ and ‘low narcissism’ prototypes have six faces in common. Similarly, the ‘low psychopathy’ and ‘low Machiavellism’ prototypes share six faces in common, so do the ‘low narcissism’ and ‘low Machiavellism’ prototypes.

**Figure 2.**
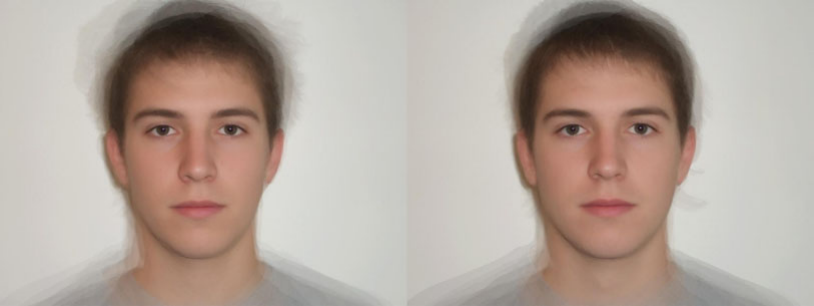
Male prototypes examples from the Faceaurus (Holtzman, 2018). High agreeableness on the left and low psychopathy on the right. These two prototypes have seven faces (out of 10 faces) in common.

In the Faceaurus dataset, there is some overlap between the prototypes, as the same faces appear in several different prototypes (if we include the Dark Triad and the Big Five only, a male face appears in five prototypes on average, and up to eight prototypes), and some prototypes use similar sets of faces (up to 70% of faces in common). As a result, a prototype for one personality trait is often not independent from the prototype for another personality trait (see Figure 1 for Low Dark Triad male prototypes). This overlap is the result of both the correlation between some personality traits and the small size of the original sample (*N* = 33 for male faces). This raises two issues: (1) a few individual faces may be responsible for most of the results, even across traits, which makes the results less generalisable (see the ‘External validity’ section); (2) the observations made with one pair of prototype might not be independent of the observations made with another pair, and this non-independence is impossible to detect without going back to the original dataset used to create the prototypes; and (3) resulting from (1) and (2) combined, it is hard to know exactly what is being measured or evaluated by the observers. Given that some of these prototypes have 70% of faces in common (e.g. the high agreeableness and low psychopathy prototypes have seven faces out of 10 in common, see Figure 2), it is difficult to disentangle if participants are detecting high agreeableness or low psychopathy, for example. Although Holtzman explicitly acknowledged this limitation of the Faceaurus dataset (Holtzman, 2011: 650), only one other publication in our dataset (Alper et al., 2021b) mentioned the fact that the same faces appear in multiple different prototypes.

#### Different studies using the same stimuli

In August 2021, we found 21 published papers using prototypes from the Faceaurus to create their facial stimuli, and this number continues to grow. This intensive use of the same dataset adds another layer of dependence in the units of information. Indeed, additional studies finding similar results contribute to giving a false impression that these results are robust. The problem is that these new studies are not independent replications (in that sense they are ‘pseudo-replications’, although this will not usually fall into the classical definition of pseudoreplication; Hurlbert, 1984), as they do not use new stimuli datasets. Thus, any new study using the same prototypes only gives us more confidence in the fact that new observers react similarly toward this specific stimuli set (Westfall et al., 2015). It does not, however, give us any additional confidence in the fact that the personality trait can be detected in faces in the population, as it is often assumed. Even when two different studies use different prototypes from the Faceaurus, they might still not represent an independent data point because of the overlap in faces used across prototypes (see above). This leads to inflated confidence in the fact that different personality traits can be detected in faces, as different studies (which could be published in different papers by the same or different authors) using different prototypes from the same dataset give the false impression that they contribute independent data.

While the authors of the publications in our dataset mentioned that they used prototypes from a previous study, this was never mentioned as a limitation, and future replications using new stimuli is suggested as a future direction in only one publication (one out of 21). This is particularly intriguing as the publications we reviewed did sometimes acknowledge the need for replication, but exclusively applied this statement to observers (e.g. Lyons & Blanchard, 2016: 42), and not to the stimuli set used.

## Discussion

Computer graphic techniques that allow the creation of realistic facial stimuli have been extensively used to explore the role of faces and face perception in social interactions and human behaviour. In particular, the prototyping (also known as the averaging or composite) technique is commonly used to investigate people's ability to detect personality traits based on static facial features. The prototype method is popular in face research because it has several advantages. The main benefit of using prototypes is that it can increase statistical power by decreasing the within-group variability (the standardisation resulting from averaging faces experimentally controls for confounding facial information) and potentially by increasing the difference between the compared groups by increasing the degree of facial transformation.^3^ A secondary but non-trivial advantage of the prototype method is the protection of the anonymity of the subjects (individual faces stop being identifiable when enough faces are averaged to create the prototype). This anonymity can help reach groups that would usually not participate in face research because they are not comfortable with the idea of having their picture displayed, which ultimately helps to collect more diverse samples with wider ranges for our traits of interest.

In this paper, we examined some methodological issues linked to the application of this technique by conducting a systematic review on all the studies using a specific dataset of prototype faces (the Faceaurus, Holtzman, 2018) as a case study. To begin, we argue that lack of external validity is a serious issue in studies using prototype faces. This lack of external validity is mainly induced by the small and non-representative sample of faces used to create prototypes, which are nevertheless argued to represent a whole population of individuals (e.g. all extraverted men in the population of interest). The lack of external validity in studies using prototypes both generates and is amplified by the non-independence of units of information encountered at several levels: at the stimuli production level (as the same individual faces are used to create different prototypes and the same prototypes are used to produce several variants of facial stimuli), at the study level (as a unique prototype per group is used) and finally at the research field level (as the exact same prototypes are repeatedly used across different studies). The main consequence of the combined effect of the lack of external validity and non-independence in the units of information is an overestimation of the replicability and generalisability of the results from studies using the prototype method.

Our systematic review also revealed that this research subfield demonstrates a worrying lack of awareness of these major limitations, as well as a lack of transparency regarding the methods used to create the facial stimuli. While various limitations are acknowledged (e.g. the need to recruit a more diverse sample of observers), the above-discussed problems of external validity and non-independence are largely ignored in the publications included in our literature review. Although we did not expect the authors of these publications to use the specific terms of ‘pseudoreplication’ or ‘external validity’ as this terminology varies between different academic fields, we did expect them to discuss, for example, the limited generalisability of their results based on the small size of the sample used to create the prototypes.

Although we focused on the Faceaurus dataset, it is important to note, however, that the issues described in this paper are not limited to studies using this specific dataset but rather encompass the whole field of face research using prototypes. Indeed, using one single prototype per group seems to be the norm rather than the exception in studies using prototypes, and prototypes are often created with small samples of 15 or fewer individual faces (for example: see Antar & Stephen, 2021; Boothroyd et al., 2008; Little & Perrett, 2007; Penton-Voak et al., 2006). Similarly, although we covered several methodological issues linked to the prototype method in this paper, this is not an exhaustive list of issues in this field of research. In particular, some of the methods used in face perception (e.g. face prototypes and the two-alternative forced-choice design) have been criticised for reasons other than the one presented here, including their lack of ecological validity and their proneness for inflating effect sizes and producing false-positive results (DeBruine, 2020; Jones & Jaeger, 2019; Pollet & Little, 2017).

What are the solutions to overcome the issues we discussed? To begin with, we argue that future studies should create and use novel datasets of prototypes. Indeed, we urgently need true replications (e.g. Shiramizu et al., 2019) to test whether previous results generalise beyond a specific set of stimuli before building follow-up hypotheses assuming the robustness of personality detection based on faces. Simply put, replications should include not only new samples of observers but also different individuals whose faces will be used to create new prototypes and facial stimuli (Linden & Hönekopp, 2021; Westfall et al., 2015). This will address the issue of non-independence of results across studies. Ideally, the new prototypes will be created based on subsamples including more than 10 individuals (e.g. the group representative of extraverts should include more than 10 individuals scoring high on extraversion) to increase their external validity. Of course, this will require full samples that include more than 50 individuals (the exact minimal sample size required remains to be calculated for each specific case). In addition, some consideration should be given to the range of the measured trait. For example, if the theoretical framework involves behaviours related to individuals with extreme personality traits, the sample used to create the prototypes should include individuals with these extreme personality traits. This could be achieved by increasing the full original sample size or by targeted sampling at the extremes of the population's distribution. Moreover, to avoid simple pseudoreplication, more than one prototype per group should be created and presented to observers (e.g. several prototypes representative of individuals scoring high on extraversion, as well as several prototypes representative of individuals scoring low on extraversion; Chouinard-Thuly et al., 2017; Kroodsma et al., 2001; Wiley, 2003). The ratings or choices of the observers should then be analysed using multilevel modelling to take into account the non-independence of the responses and avoid the overestimation of the degrees of freedom (e.g. Chaves, 2010; Millar & Anderson, 2004; Pollet et al., 2015; but see Arnqvist, 2020). An extension of this solution is to use the individual faces as stimuli, which is a valid alternative to prototypes that needs to be considered when designing a study.

Although there is no ideal research design, there is an ideal way to report studies (Wiley, 2003). Two features are important for a good report: the first one is to provide enough information to allow the reader to fully understand the details of the methods used (and to allow potential replications of the study), and the second is to be aware of and to acknowledge the compromises made and the limitations inherent to any research design. Thus, future studies using facial prototypes should be more transparent when reporting the methods used and discussing their limitations. Basic information regarding the creation of the visual stimuli should be reported, including the number of faces averaged for each prototype, the size of the full sample from which the faces were selected from and the range of the measured trait (e.g. extraversion score), as it is necessary for the interpretation of the results. Although some methodological issues in this research field can be easily fixed (e.g. using more than one prototype per group), some methodological limitations are probably unavoidable (e.g. limited sample sizes), simply because of time and resource constraints. It is crucial, therefore, to clearly acknowledge any limitations and include claims of external validity to know which conclusions can be safely drawn from the results and which are more speculative, as well as to determine the focus for any follow-up studies (Simons et al., 2017).

Why should we care about these methodological issues and limitations? The first obvious reason is that we want rigorous methods to provide reliable results, independently of the research question. The second reason is that facial prototype methods are used to explore a fascinating but controversial topic. Indeed, the idea that personality traits can be accurately detected in static facial features (a ‘kernel of truth’ in facial inferences) is a highly debated topic (Bonnefon et al., 2015; Todorov et al., 2015) raising a strong public interest (Foo et al., 2021). Physiognomy has a bad reputation in Psychology, and this is largely well deserved: most ‘studies’ in the area have been resolutely unscientific, leading physiognomists to be dismissed as charlatans (Penton-Voak et al., 2006), even though there are also some reasonable theoretical claims underlying a possible association between facial appearance and individuals’ personality or behaviour (Penton-Voak et al., 2006). Thus, when testing such controversial ideas, it is crucial to use rigorous methods.

Even though we have painted a rather gloomy picture of this field of research, we believe there are some encouraging signs as well. Notably, Shiramizu et al. (2019) noticed that most studies focussing on facial correlates of the Dark Triad were using the same dataset (the Faceaurus), and they conducted a (true) replication by generating a new stimulus set. Similarly, Jaeger et al. (2021) conducted preregistered replications of studies exploring the perception of extraverted-looking individuals^4^ where they used new stimuli sets in addition to the Faceaurus dataset, and discussed various kinds of limitations. Holtzman added the link to a blog post discussing the false-positive inflation issue in studies using face prototypes (DeBruine, 2020) on his website https://nickholtzman.com/faceaurus/ (‘Faceaurus’, 2016). Finally, in some studies, the faces used to create the prototypes were selected among larger full samples than the one used for the Faceaurus (for example, see Jones et al., 2012; Penton-Voak et al., 2006). To conclude, we believe that the prototype method is promising (beyond the topic of personality detection and even outside Psychology), if well used, and we hope that this paper can help us move toward improvement in this research area.

## Data Availability

The dataset for the systematic review and the associated R script are openly available on the Open Science Framework at https://osf.io/t2kz3

## Footnotes

1 A high number is not so surprising here, as one of the inclusion criteria for our systematic review was the use of the Faceaurus dataset. What is surprising, however, is that none of these studies used triangulation through the inclusion of additional stimuli (e.g. different prototypes; individual faces; voices; etc.).

2 Owing to missing data, these sample sizes are actually smaller for some traits (e.g. *N* = 28 men for agreeableness).

3 An obvious drawback is the lack of ecological validity of such stimuli.

4 This paper was not included in our final dataset, as it was not published in a peer-reviewed journal when we conducted this literature review.
